# An Improved P-Type Doped Barrier Surface AlGaN/GaN High Electron Mobility Transistor with High Power-Added Efficiency

**DOI:** 10.3390/mi12091035

**Published:** 2021-08-28

**Authors:** Hujun Jia, Xiaowei Wang, Mengyu Dong, Shunwei Zhu, Yintang Yang

**Affiliations:** School of Microelectronics, Xidian University, Xi’an 710071, China; boywangxw@126.com (X.W.); mengyudong@stu.xidian.edu.cn (M.D.); swzhu@stu.xidian.edu.cn (S.Z.); ytyang@xidian.edu.cn (Y.Y.)

**Keywords:** GaN, HEMT, PDBS, power-added efficiency

## Abstract

An improved P-type doped barrier surface AlGaN/GaN high electron mobility transistor with high power-added efficiency (PDBS-HEMT) is proposed in this paper. Through the modelling and simulation of ISE-TCAD and ADS software, the influence of the P-type doped region on the performance parameters is studied, and the power-added efficiency (PAE) obtained and effectively improved is further verified. The drain saturation current and the threshold voltage of PDBS-HEMT has no major change compared with the traditional structure; the peak transconductance decreases slightly, but the breakdown voltage is significantly enhanced. Furthermore, the gate-source capacitance and gate-drain capacitance are reduced by 14.6% and 14.3%, respectively. By simulating the RF output characteristics of the device, the maximum oscillation frequency of the proposed structure is increased from 57 GHz to 63 GHz, and the saturated output power density is 10.9 W/mm, 9.3 W/mm and 6.4 W/mm at the frequency of 600 MHz, 1200 MHz and 2400 MHz, respectively. The highest PAE of 88.4% was obtained at 1200 MHz. The results show that the PDBS structure has an excellent power and efficiency output capability. Through the design of the P-type doped region, the DC and RF parameters and efficiency of the device are balanced, demonstrating the great potential of PDBS structure in high energy efficiency applications.

## 1. Introduction

As one of the third-generation semiconductors, Gallium Nitride has a higher critical breakdown field and wider bandgap than other materials, such as Si and GaAs, so that it can withstand a greater power density of the same size. Furthermore, the higher electron saturation velocity promoted the frequency characteristics and the output current, and the better anti-radiation characteristics enhanced the stability of the device [1]. For AlGaN/GaN heterojunction, because of the spontaneous polarization and piezoelectric polarization effects, the high concentration and high mobility 2DEG can be generated at the interface of the heterojunction. Therefore, GaN-based power devices exhibit unprecedented application prospects of microwave power amplification [2]. In the traditional AlGaN/GaN HEMT design, people focus on improving the performance parameters. For example, the breakdown voltage is strongly limited by the high electric field peak near the gate edge. To solve this problem, the design between the gate and drain is used to modify the charge distribution, eliminate the obvious electric field peak and make the surface electric field distribution more uniform, and the breakdown voltage is increased [3]. Therefore, a variety of methods have been proposed, such as partially etched [4] and P-type doped [5] in the AlGaN barrier layer near the gate side between the gate and drain, enhancement mode device with par-tially GAN cap layer [6], DRBL structure with a double recess barrier layer on both sides of the gate [7], and changing the distance between the gate and drain [8].

However, with high efficiency and low energy consumption gradually becoming an important direction, the research on the power-added efficiency of devices becomes more and more important, but the related reports are very rare. In only a few studies, it has been found that among the many electrical parameters of the device, the improvement of power-added efficiency caused by the structural change near the drain side is not obvious [9,10]. Therefore, in this article, an improved P-type doped barrier surface AlGaN/GaN high electron mobility transistor (PDBS-HEMT) with high power-added efficiency is proposed. The ISE_TCAD software is used for device modelling and simulation, and the EE HEMT model in the ADS software is used for RF output characteristics’ simulation. The drain saturation current, threshold voltage, trans-conductance, capacitance and other parameters of PDBS-HEMT are studied, and the power and efficiency are analyzed under different work conditions. It is proved that the P-type doped structure on the surface of the barrier layer is effective for the improvement of PAE, and the influence mechanism of various parameters on PAE is discussed.

## 2. Device Structure and Simulation Method

**Figure 1:**
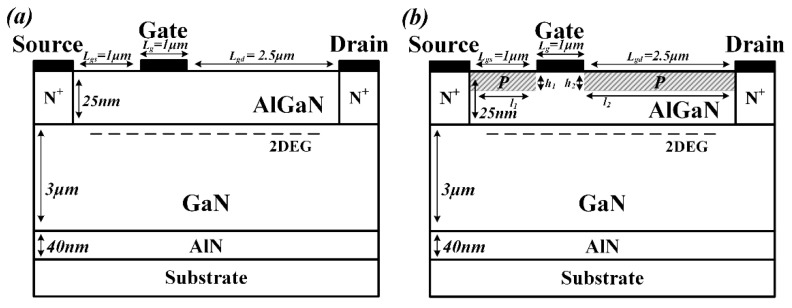
Schematic cross−sections of the (**a**) Con−HEMT, (**b**) PDBS−HEMT.

The schematic cross-sectional view of the Con-HEMT and the PDBS-HEMT are depicted in Figure 1a,b. To establish a meaningful comparison, they are designed with the same doping level and size as far as possible. Both structures include a 25 nm Al_0.32_Ga_0.68_N barrier layer, a 3 µm GaN buffer layer, a 40 nm AlN nuclear layer and a SiC substrate layer. The source region and drain region on the left and right sides of the barrier layer are heavily doped with a concentration of 1 × 10^20^ cm^−3^ of N-type. The distance between gate-source and gate-drain is 1 µm and 2.5 µm, and the length of the gate, source and drain are all 1 µm. To simulate the background carriers, the GaN buffer layer is doped with a concentration of 1 × 10^15^ cm^−3^ of N-type. The Schottky contact metal work function of the gate is 1.0 eV. The difference is the two P-type doped regions on the AlGaN barrier layers surface, which are P-type doped with the concentration of 1 × 10^16^ cm^−3^, where *l_1_* = *Lgs* = 1 µm, *l_2_* = *Lgd* = 2.5 µm and *h_1_ = h_2_* = 0.01 µm. It is worth noting that they can be manufactured with the same procedure reported in the references [4,5].

**Figure 2:**
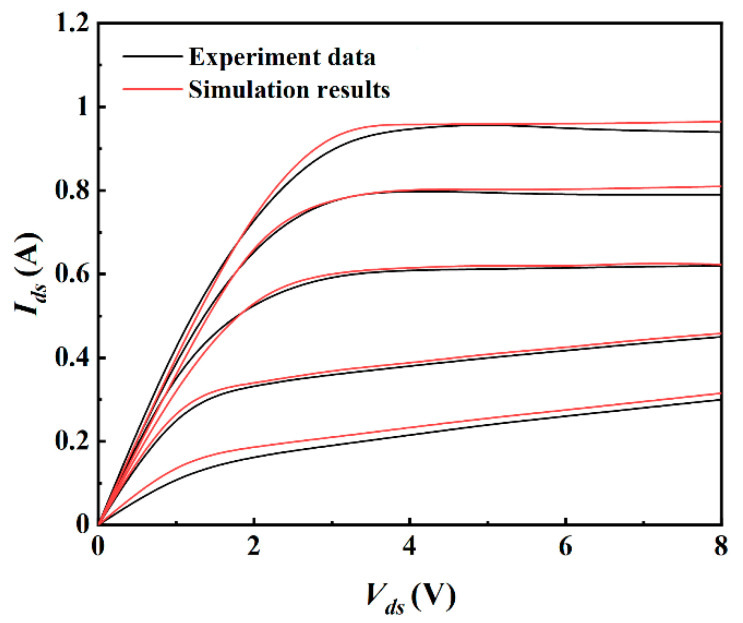
Comparison of experimental data and simulation data on *I_ds_*−*V_ds_.*

The ISE_TCAD software is used in two-dimensional device simulation. The simulator is calibrated with experimental data in reference [11], and the agreement between experimental data and simulation results is obtained as shown in Figure 2.

To get more realistic results, the temperature of 300 K is maintained during the simulation in the ISE_TCAD. Several models are activated, including the basic Poisson equations, Schrödinger–Poisson coupled equations, drift-diffusion equations, the Generation and Recombination model (SRH and Auger), the Incomplete ionization model and the Mobility model (Doping Dep, Enormal, and High Fieldsat). The criterion of breakdown was BreakCriteria {Current (Contact = “Drain” Absval = 1 × 10^−4^)}, and the others parameters are set by referring to the parameters in reference [12]. After simulation, these parameters are effective and practical. The EE_HEMT model is used in ADS, in which the sensitive parameters of PAE mainly include threshold voltage (*V_t_*), maximum transconductance (*G_mmax_*), gate-source capacitance (*C_gs_*_)_ and gate-drain capacitance (*C_gd_*). Through the extraction of these PAE sensitive parameters in the device simulation, we can build an effective PAE measurement model in ADS [13,14].

## 3. Results and Discussion

### 3.1. DC Characteristics

**Figure 3:**
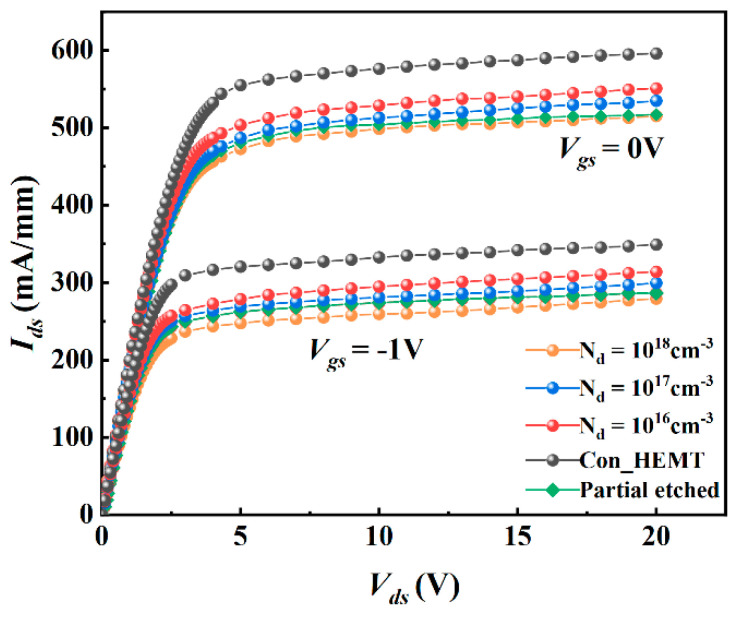
*I_ds_*−*V_ds_* of the proposed structure with different doping concentration.

Figure 3 shows the current output characteristic (*I_ds_-V_ds_*) when the gate bias is −1 V and 0 V. It can be seen that due to the lead-in of the P-type doped region in the barrier layer, the drain saturation current (*I_dsat_*) of the device decreases slightly. When $V_{gs}$ = 0 V, for PDBS-HEMT with a doping concentration of 1 × 10^16^ cm^−3^, *I_dsat_* is 551 mA/mm, which is slightly less than 596 mA/mm of the traditional structure. With the increase of doping concentration, the *I_dsat_* of the proposed structure decreases continuously. When the doping concentration is 1 × 10^18^ cm^−3^, the *I_dsat_* of the proposed structure is 516 mA/mm. The insertion of the P-type doped region reduced the net donor impurity density in the barrier and the net electron density in the channel. Figure 3 includes the simulation results of the structure with a partially etched barrier layer with the same size as the proposed structure. Compared with the change of etching in the barrier layer, inserting a P-type doped region can reduce the current loss to the greatest extent. That is because the area density of 2DEG in the AlGaN/GaN HEMT channel is obviously proportional to the thickness of the AlGaN barrier layer.

**Figure 4:**
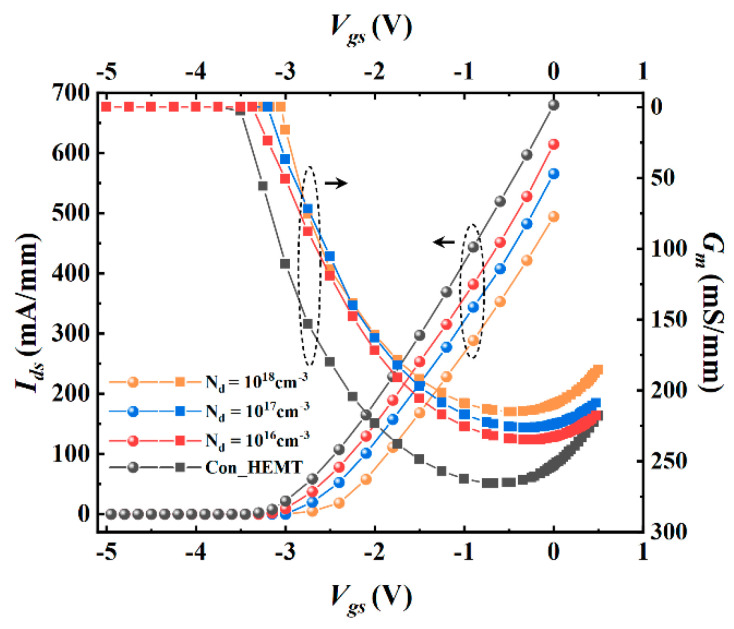
*I_ds_*−*V_gs_* and *G_m_*−*V_gs_* of the proposed structure with different doping concentration.

Figure 4 shows the transfer characteristic (*I_ds_* − *V_gs_*) and transconductance (*G_m_* − *V_gs_*) of the device. The threshold voltage of the PDBS structure is −3.3 V, while that of the traditional structure is −3.5 V. The barrier layer under the gate of the proposed structure is the same as the traditional structure, so the threshold voltage is similar for the two structures. The threshold voltage for the normally-on device is the voltage required on the gate when the current in the channel is pinched off. The decrease of 2DEG concentration in PDBS-HEMT makes it easier to apply a negative bias to the gate to deplete 2DEG in the channel, so the absolute value of the threshold voltage decreases.

The physical definition of transconductance is the first derivative of drain output current and input voltage, which reflects the dependence of drain current on the gate-source voltage at a fixed drain-source voltage.

(1) $$g_{m}=\frac{\partial I_{ds}}{\partial V_{gs}}|{}_{V_{ds}=const}$$

The doped region improves the electric field distribution in the barrier layer and weakens the control effect of the drain-source voltage on the current in the channel, but the influence on the modulation effect of the electric field is limited. The peak transconductance of the proposed structure is 230 mS/mm, which is 13.2% lower than 265 mS/mm of traditional structure. This means that the traditional structure has a larger drain saturation current under the same gate voltage bias, and it can be seen that the increase of doping concentration further weakens the control of the drain-source voltage on current.

**Figure 5:**
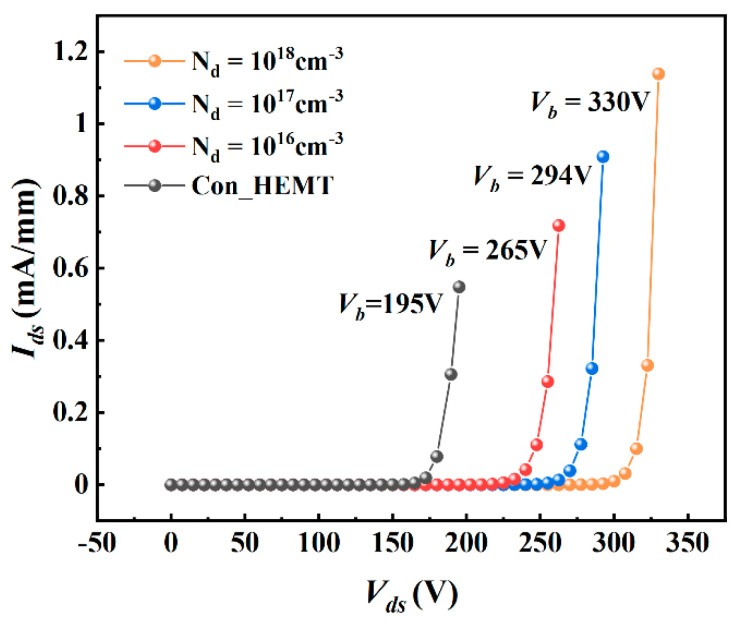
Simulated breakdown characteristics of the proposed structure with different doping concentration.

**Figure 6:**
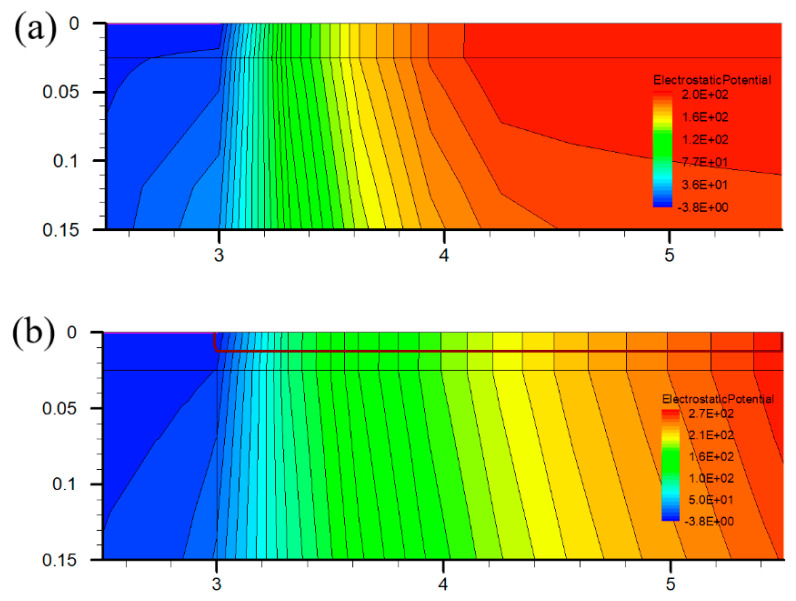
Distributions of the electrostatic potential of (**a**) Con−HEMT and (**b**) PDBS−HEMT.

Figure 5 shows the breakdown characteristic. The higher the concentration of the P-type doped region, the higher the breakdown voltage. The breakdown voltage of the proposed structure with 1 × 10^18^ cm^−3^ is 330 V, which is 69% higher than 195 V of the traditional structure. It can be seen from the distribution of the electric field in Figure 6 that the P-type doped region between the gate and drain improves the potential distribution in the barrier layer, alleviates the electric field crowding and improves the withstand voltage of the device. It is also helpful to reduce the surface leakage current and current collapse effect due to the alleviation of electric field crowding.

### 3.2. AC Characteristics

**Figure 7:**
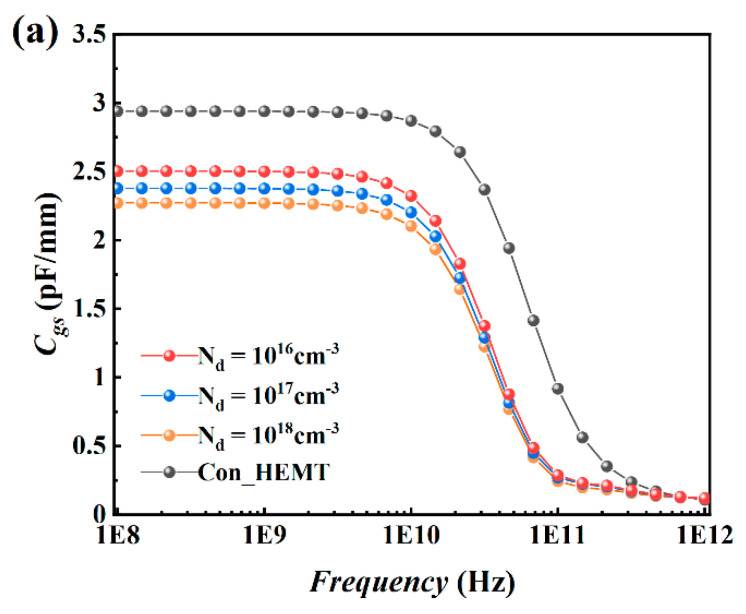

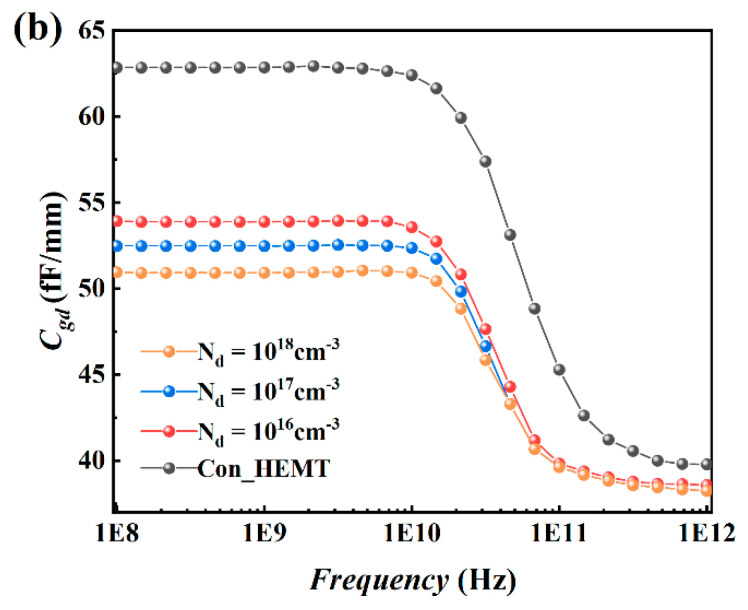
(**a**) *C_gs_*−*F_re_q* and (**b**) *C_gd_*−*F_req_* of the proposed structure with different doping concentration at $V_{gs}$ = 0 V and $V_{ds}$ = 20 V.

**Figure 8:**
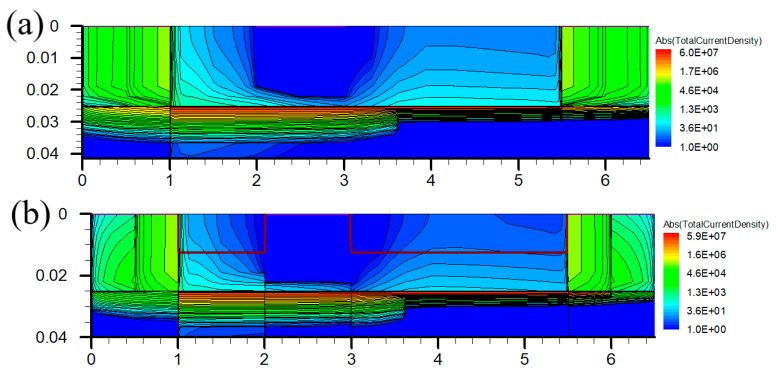
Distributions of the current density of (**a**) Con-HEMT and (**b**) PDBS-HEMT.

Figure 7 shows the curve of *C_gs_* and *C_gd_* to frequency under the conditions of $V_{gs}$ = 0 V and $V_{ds}$ = 20 V for PDBS structure with different doping concentrations. The *C_gs_* of the PDBS structure is 2.5 pF/mm and the *C_gd_* is 53.9 fF/mm, which are 14.6% and 14.3% lower than the traditional structure (2.93 pF/mm and 63 fF/mm). Furthermore, as the doping concentration increases, the capacitance is further reduced. It can be seen from the current density distribution in Figure 8 that the doped region located between the gate-source and the gate-drain effectively suppresses the extension of the depletion layer under the gate to both sides, which increases the distance from the gate to the lower surface of the depletion layer in the vertical direction, so the capacitance decreases significantly.

**Figure 9:**
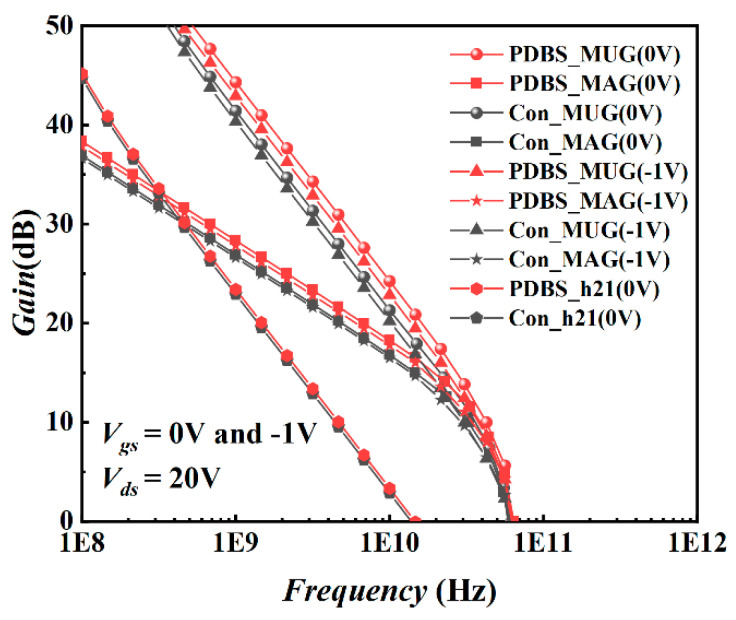
Small−signal high-frequency characteristics of PDBS−HEMT and Con−HEMT.

Equation (2) is the calculation formula of the cut-off frequency of the HEMT device. It can be seen from the formula that the cut-off frequency is simultaneously restricted by the transconductance and the gate-source capacitance. For PDBS structure, the cut-off frequency does not change greatly due to the decrease of transconductance and capacitance at the same time, which is increased from 14.3 GHz of the traditional structure to 14.6 GHz. Figure 9 shows the small-signal high-frequency characteristic of the two structures under the conditions of $V_{gs}$ = 0 V(−1 V) and $V_{ds}$ = 20 V, in which the h21 is the small signal current gain of the device, the maximum available gain (MAG) is the maximum gain and the MUG is the unilateral power gain of the device. When the h21 drops to 0 dB, the cut-off frequency of the proposed structure is almost the same as the Con-HEMT, which also verifies the calculation result above. When the unilateral power gain (MUG) and the maximum available gain (MAG) drop to 0 dB, the maximum oscillation frequency (*f*_max_) of the PDBS structure is 63 GHz, which is 10.5% higher than the 57 GHz of the traditional structure. It can be seen from Equation (3) that due to the PDBS structure, it has a smaller gate resistance, and the doped region increases the drain-source resistance to a certain extent, and thereby the *f*_max_ is increased. It can also be seen from Figure 9 that the MUG and MAG of PDBS structure have increased by 2.4 dB and 1.2 dB compared with the traditional structure at the same frequency on the $V_{gs}$ = 0 V, respectively. When the $V_{gs}$ = −1 V, the MAG and the MUG decreased slightly, but the *f*_max_ does not show an obvious change. This means that the PDBS structure has greater power conversion capability and higher output efficiency. Some parameters of the PDBS structure are summarized in Table 1.

(2) $$f_{t}\approx \frac{g_{m}}{2\pi C_{gs}}$$

(3) $$f_{\max}\approx \frac{f_{t}}{2}\sqrt{\frac{R_{ds}}{R_{g}}}$$

### 3.3. Energy and Efficiency

**Figure 10:**
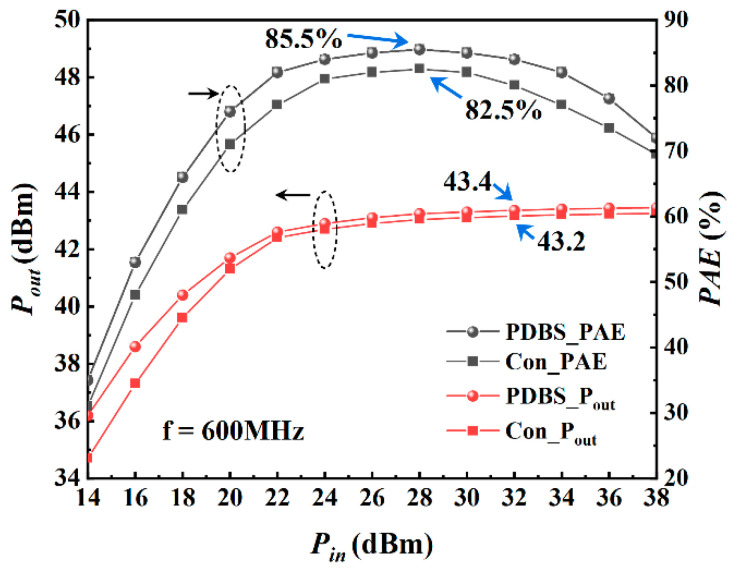
Large-signal performance of both structures at 600 MHz with $V_{gs}$ = −4 V, $V_{ds}$ = 20 V.

**Figure 11:**
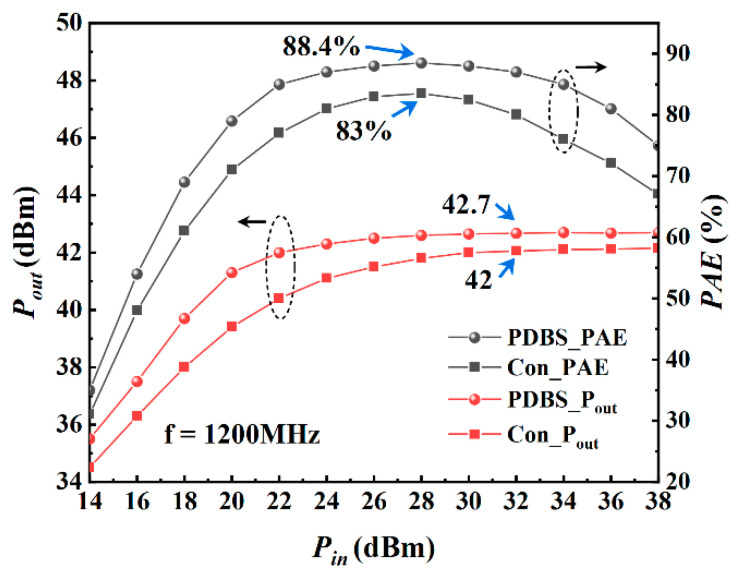
Large-signal performance of both structures at 1200 MHz with $V_{gs}$ = −4 V, $V_{ds}$ = 20 V.

**Figure 12:**
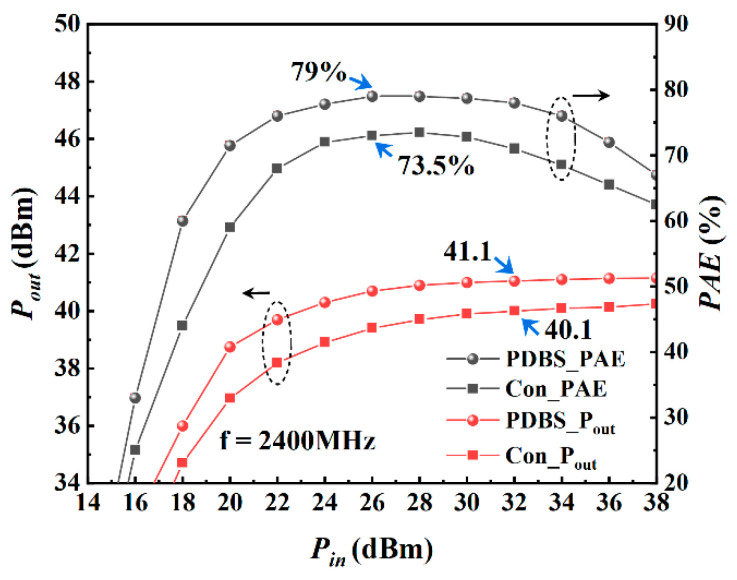
Large-signal performance of both structures at 2400 MHz with= −4 V, $V_{ds}$ = 20 V.

Figure 10 shows the results of large-signal simulation of the device under the condition of $V_{gs}$ = −4 V, $V_{ds}$ = 20 V and frequency of 600 MHz. When the input power reaches 28 dBm, the maximum PAE of the proposed structure is 85.5%, while the traditional structure is 82.5%. At this time, the saturated output power is 43.4 dBm, the saturated output power density is 10.9 W/mm, and the saturated power gain is 11.4 dB. Figure 11 shows the simulation results at 1200 MHz. As can be seen from the figure, when the input power reaches 28 dBm, the maximum PAE is 88.4%, while the traditional structure is 83%. At this time, the saturated output power is 42.7 dBm, the saturated output power density is 9.3 W/mm, and the saturated power gain is 10.7 dB. Figure 12 shows the simulation results at 2400 MHz. It can be seen that the power and efficiency output capability of the device is weakened at higher working frequency, but as expected, the proposed structure has higher power and efficiency output capability than the traditional structure in the entire frequency band, and the saturated area of output power is broadened. At 2400 MHz, when the input power reaches 26 dBm, the maximum PAE is 79%, the saturated output power is 41.1 dBm, the output power density is 6.4 W/mm and the power gain is 9.1 dB.

PAE is the ratio of the difference between the output and input power and the DC bias power, which reflects the power amplification capability of the device. The definition is as Equation (4):

(4) $$PAE=\frac{P_{out}-P_{in}}{P_{dc}}=\frac{P_{in}}{P_{dc}}(G-1)$$

where *P_out_* is the output power, *P_in_* is the input power, *P_dc_* is the DC bias power and *G* is the power gain.

It can be seen from Equation (4) that PAE is affected by a variety of factors. Under the same input, the smaller the DC power consumption, the larger the power gain and the larger the PAE. The change of the device structure will cause the performance parameters to change, then affect the output power and gain, and ultimately lead to the change of PAE. The absolute value of the threshold voltage of the PDBS structure is slightly smaller than that of the traditional structure, so the gate-source voltage across which the device rises from the off state to the maximum transconductance state becomes smaller, which can significantly enhance the amplification capability of the device and improve the power gain. Furthermore, the smaller the threshold voltage brought a smaller DC power consumption, and thereby the PAE ios increased. GaN-based devices can work at a higher frequency, but the effect of various capacitive and inductive reactance inside the device is more sensitive to frequency. The gate-source capacitance (*C_gs_*) and gate-drain capacitance (*C_gd_*) are the main sources of parasitic capacitance inside the device, the larger the internal capacitance of the device, the greater the energy loss of charging and discharging by gate voltage, which seriously damages the efficiency of the device, so the reduction of capacitance is also one of the reasons for the increase of PAE. The comparison of output characteristics of the PDBS-HEMT and Con-HEMT at different frequencies are summarized in Table 2.

## 4. Conclusions

In this paper, the DC and RF output characteristics of the P-type doped barrier surface AlGaN/GaN high electron mobility transistor (PDBS-HEMT) are studied. The results show that the PDBS structure has excellent power and efficiency output capability through the lead-in of the P-type doped region. The drain saturation output current and maximum transconductance of the proposed structure are 534 mA/mm and 230 mS/mm, respectively, which are 10% and 13.2% lower than the traditional structure. The breakdown voltage is 265 V and increases with the doping concentration. Compared with the traditional structure, the maximum oscillation frequency and cut-off frequency of the device are slightly improved. The maximum saturation output power density is 10.9 W/mm, the maximum saturation power gain is 11.4 dB and the maximum PAE is 88.4%. In general, the experiment proves that the DC and RF parameters of AlGaN/GaN HEMT can be balanced by an appropriate trade-off, which provides a theoretical basis and design method for further design of a high-efficiency RF power amplifier.

## Figures and Tables

**Table 1:** Parameters of the PDBS-HEMT and Con-HEMT.

Parameters	PDBS-HEMT	Con-HEMT
*Id_sat_* (mA/mm)	534	596
*V_t_* (V)	−3.4	−3.5
*G_mmax_* (mS/mm)	230	265
*V_b_* (V)	265	195
*C_gs_* (pF/mm)	2.5	2.93
*C_gd_* (fF/mm)	53.9	63

**Table 2:** Comparison of output characteristics of the PDBS-HEMT and Con-HEMT at different frequencies.

Parameters	PDBS-HEMT			Con-HEMT		
	Frequency					
	600 MHz	1200 MHz	2400 MHz	600 MHz	1200 MHz	2400 MHz
Power density	10.9 W/mm	9.3 W/mm	6.4 W/mm	10.4 W/mm	9.3 W/mm	5.1 W/mm
Gain	11.4 dB	10.7 dB	9.1 dB	11.2 dB	10.0 dB	8.1 dB
PAE (max)	85.5%	88.4%	79%	82.5%	83.0%	73.5%

## Data Availability

Not applicable.
